# Reproductive Performance of Female Rabbits Inseminated with Extenders Supplemented with GnRH Analogue Entrapped in Chitosan-Based Nanoparticles

**DOI:** 10.3390/ani13101628

**Published:** 2023-05-12

**Authors:** Maria Pilar Viudes-de-Castro, Francisco Marco Jimenez, José Salvador Vicente

**Affiliations:** 1 Centro de Investigación y Tecnología Animal, Instituto Valenciano de Investigaciones Agrarias (CI-TA-IVIA), Polígono La Esperanza No. 100, 12400 Segorbe, Spain; 2 Instituto de Ciencia y Tecnología Animal, Universitat Politècnica de València, 46022 Valencia, Spain; fmarco@dca.upv.es (F.M.J.);

**Keywords:** rabbit, artificial insemination, nanoencapsulation, chitosan, dextran sulphate, alginate

## Abstract

**Simple Summary:**

Ovulation in female rabbits is induced via the sensory stimulation associated with mating. Consequently, when artificial insemination is used, as there is no stimulation via coitus, an analogue of the hormone responsible for ovulation induction must be used, which could be performed intramuscularly, intravenously, or intravaginally. Addition of the hormonal analogue to the seminal dose, for intravaginal administration, is the least stressful way to induce ovulation in does. However, due to the high level of enzymes present in rabbit seminal plasma and the poor permeability of the vaginal mucosa, the amount of analogue required for successful ovulation induction via the vaginal route may be 20–30 times the dose needed for the intramuscular route. Protection of the analogue via nanoencapsulation can help overcome enzyme degradation and improve its bioavailability. The efficacy of two extenders with chitosan–dextran sulphate or chitosan–alginate nanoparticles entrapping the hormone analogue will be studied. The main aim of the current work is to assess the effect of both encapsulation systems on reproductive performance after insemination. Results showed that both nanoencapsulation systems used here are an efficient way of intravaginal ovulation induction, allowing a reduction in the concentration of the hormone used in artificial insemination to four micrograms per female without affecting fertility and prolificacy.

**Abstract:**

Rabbit is a reflexively ovulating species. Accordingly, in the practice of artificial insemination (AI) ovulation must be induced via exogenous GnRH (Gonadotropin-Releasing Hormone) administration, which may be performed intramuscularly, subcutaneously, or intravaginally. Unfortunately, the bioavailability of the GnRH analogue when added to the extender is lower due to the proteolytic activity in the seminal plasma and the poor permeability of the vaginal mucosa. The aim of the study was to refine the practice of AI practice in rabbits by replacing parenteral GnRH analogue administration (subcutaneous, intravenous, or intramuscular injection) with intravaginal application, while reducing its concentration in the diluent. Extenders containing the buserelin acetate in chitosan–dextran sulphate and chitosan–alginate nanoparticles were designed and 356 females were inseminated. Reproductive performance of females inseminated with the two experimental extenders, receiving 4 μg of buserelin acetate intravaginally per doe, was compared with that in the control group, the does of which were inseminated with the extender without the GnRH analogue and induced to ovulate with 1 μg of buserelin acetate administered intramuscularly. The entrapment efficiency of the chitosan–dextran sulphate complex was higher than that of chitosan–alginate. However, females inseminated with both systems showed similar reproductive performance. We conclude that both nanoencapsulation systems are an efficient way of intravaginal ovulation induction, allowing a reduction in the level of the GnRH analogue normally used in seminal doses from 15–25 μg to 4 μg.

## 1. Introduction

Artificial insemination (AI) is a highly efficient assisted reproductive technology that has become a common practice in rabbit farms. When practicing AI in this species, as rabbit is an induced ovulator, it is necessary to use gonadotropin-releasing hormone (GnRH) analogues to trigger ovulation. GnRH could be administrated intramuscularly, subcutaneously, intravenously, or intravaginally. The most common method used is intramuscular administration of GnRH, but the intravaginal route can be considered the least stressful way to induce ovulation, making it a welfare-orientated method that has clear advantages, as it is a non-invasive route and reduces both farmers’ labour and handling times. Hence, rabbit AI with GnRH-supplemented extenders increases the welfare of rabbit insemination procedures. The abundant blood supply and large vaginal surface area of the rabbit doe allow a rapid absorption of low molecular weight drugs [1,2]. Nevertheless, vaginal absorption of synthetic GnRH analogues is less efficient than that via parenteral analogues (roughly five times less) and was found to result in 20% bioavailability following combination with organic acids [3]. On the other hand, it was found that 50% of the proteins identified in the rabbit seminal plasma proteome have catalytic activity [4]. One of these proteins is aminopeptidase B, an enzyme with a crucial role in rabbit AI when GnRH is added to the diluent, since it is capable of degrading GnRH analogues when present. The absorption of GnRH by vaginal mucosa is influenced by several factors, such as proteases present in the seminal plasma [5,6], extender composition [7], the status of the vaginal mucosa [8,9,10] and the GnRH analogue used [11]. Consequently, the bioavailability of GnRH could be variable and much lower when its application is intravaginal. Therefore, to achieve fertility results that are like those obtained with intramuscular injection, the concentration of the GnRH analogue used in the extenders must be higher than that applied intramuscularly [6]. However, when using a GnRH analogue in the extender, it must be considered that the analogues possess greater biological activity than the GnRH does itself. The usual GnRH analogue dose used in semen extenders is from a 15- to 25-fold higher concentration than that used for intramuscular injection [12,13], using buserelin acetate and alarelin acetate, respectively. Hence, although there are clear breeding advantages of intravaginal administration of the GnRH analogue, the use of such high doses constitutes a potential risk to the health of the personnel who prepare and dispense the seminal dose, and leads to a significant increase in the cost-efficiency of this insemination procedure. Therefore, reducing the GnRH analogue concentration in rabbit insemination extenders is still a challenging task. Different approaches with which to improve the success of the vaginal route include the use of protease inhibitors [14,15], absorption enhancers, mucoadhesive polymers and GnRH carrier systems such as nanoparticles. Among these approaches, encapsulation systems that protect the peptide from enzymatic degradation are of great interest. Nanoparticles of biodegradable polymers have been extensively studied over the last few decades in pharmaceutical research for controlled drug delivery. The main advantage of using nanoparticles is their ability to load molecules and enhance their transport across mucosal surfaces [16], hence the increasing interest in nanoencapsulation for medical and pharmaceutical applications. It has been shown that mixing two polymers that are oppositely charged in an aqueous solution may spontaneously form polyelectrolyte complexes. Many different polyanions of natural origin have been used to form polyelectrolyte complexes for the design of specific drug delivery systems [17]. One of the most commonly studied polymers for drug delivery systems is chitosan (CS), a biocompatible and biodegradable amino polysaccharide of low toxicity. CS is regarded as a sustainable material, and is the only naturally derived cationic polymer. It has been widely used for developing drug delivery systems because of its excellent mucoadhesive properties [18]. In addition, CS acts as penetration enhancer [19]. Many different complexes between CS and anionic natural polymers have been researched for drug encapsulation purposes [20]. When no chemical covalent cross-linker is used, the electrostatic attraction between the cationic amino groups of CS and the anionic groups of polyanions is the main interaction leading to the formation of nanoparticles. The use of oppositely charged polymers to encapsulate drugs is a mild method, is simple to implement, and requires no chemical cross-linker. Thus, the formation of polyelectrolyte complexes is a safe and environmentally sustainable process for manufacturing materials for drug delivery applications [21]. In the case of using chemical cross-linkers to form polymer complexes, special care must be taken with regard to the possible presence of unreacted residues due to their potential toxicity. Therefore, to avoid detrimental effects of the GnRH encapsulation procedure on sperm, a mild preparation process without involving chemical cross-linkers is preferable. The most widely used anionic polysaccharides in complexation with CS are dextran sulphate (DS) and alginate (ALG). Both are biodegradable, biocompatible, easily available and non-toxic polyanions. The negatively charged carboxyl residues in ALG and the presence of sulphate groups in DS ensure strong electrostatic interactions with amino groups of CS to form complexes [20,22]. In rabbit AI, when the vaginal route is used to induce ovulation, encapsulation of the analogue could protect the hormone from enzyme degradation, allowing greater absorption. Recently, incorporation of buserelin acetate into chitosan–dextran sulphate (CS—DS) nanoparticles was carried out to study their effect on rabbit semen quality, showing enhanced acrosome integrity and no effect on motility, viability, and membrane functionality [23]. Furthermore, when the encapsulated GnRH was used in extenders supplemented with ethylenediaminetetraacetic acid disodium salt (EDTA) and bestatin, the poor stability of the GnRH analogue in the presence of seminal aminopeptidases could be overcome, allowing a reduction in the GnRH concentration in the extender without affecting the reproductive performance of the female rabbits [24]. It has also been shown that the addition of buserelin acetate-loaded CS—sodium tripolyphosphate (TPP) nanoparticles to semen extender has negative impacts on fertility [25]. Hence, is interesting to study different CS complexes as carriers of GnRH analogues to improve the intravaginal route of GnRH administration in rabbit AI. The current study aims to evaluate the efficiency of two CS-based nanoparticles as carriers of buserelin acetate, CS—DS and CS—ALG. Their intravaginal efficacy as ovulation inductors was indirectly estimated via observing in vivo reproductive performance (fertility and prolificacy) after AI.

## 2. Materials and Methods

The chemicals used in this study were purchased from Sigma-Aldrich (Merck Life Science S.L.U. Madrid, Spain), except for the buserelin acetate, which was purchased from Hoechst Marion Roussel, S.A. (Madrid, Spain), and the DS, which was purchased from Thermofisher Acros Organics (Geel, Belgium).

### 2.1. Preparation of Extenders with GnRH-Loaded CS—DS and CS—ALG Nanoparticles

CS—DS and CS—ALG nanoparticles were prepared via the coacervation method. All polymers were dissolved (0.05%) in the control medium, which consisted of a TCG extender [26] supplemented with 10 µM obestatin and 20 mM EDTA. As chitosan (CS) is poorly soluble in water, its solution was prepared under acidic conditions, so it was dissolved first in a solution of citric acid and stirred (~800 rpm) for 60 min at 50 °C before the rest of the components of the control medium were added (Trizma base, glucose, bestatin and EDTA). This solution was stirred for a further 60 min at room temperature. Incorporation of buserelin acetate into nanoparticles was achieved by dissolving the hormone in DS and ALG solutions to obtain the desired final GnRH concentration in the diluted semen (8 μg/mL). These solutions were stirred for 30 min and directly added dropwise into the chitosan solution (charge ratio of 1:4 *v/v*) to form self-assembled nanoparticles. The mixture was stirred (~600 rpm) for a further 45 min at room temperature and the pH was adjusted to 6.8.

### 2.2. In-Vitro Determination of GnRH Analogue Entrapped in the Nanoparticles

Entrapment efficiency (EE) was determined at zero time and the release study was performed via incubating experimental extenders at 5 and 37 °C. After 5 hr, individual samples were filtered (0.22 μm filters Millex^®^ GP, Millipore, Ireland), and the amount of free hormone released in the supernatants was evaluated, measuring the protein absorbance via spectrophotometry (BioPhotometer, Eppendorf AG, Hamburg, Germany) at 280 nm (*n* = 3). Then, the GnRH analogue concentration was determined with a standard calibration curve using empty CS—DS and CS—ALG nanoparticles as the control. The EE was calculated using the following equation:

(1) $$\mathrm{EE}\;\left(\%\right)=\left(\frac{\mathrm{amount}\;\mathrm{of}\;\mathrm{GnRH}\;\mathrm{added}-\mathrm{amount}\;\mathrm{of}\;\mathrm{free}\;\mathrm{GnRH}}{\mathrm{amount}\;\mathrm{of}\;\mathrm{GnRH}\;\mathrm{added}}\right)\times 100$$

### 2.3. Animals

Males and females were sexually mature New Zealand white rabbits (A line, selected for litter size at weaning since 1980). All animals were handled according to the European regulations for the care and use of animals for scientific purposes published by the directive 2010/63/EU. Animal housing and the protocols for semen collection and AI were approved by the Animal Care and Use Committee of Centro de Tecnología Animal, Instituto Valenciano de Investigaciones Agrarias. The animals were housed in flat-deck cages (40 cm wide × 100 cm long × 40 cm high), under a 16 h light and 8 h darkness photoperiod, fed a standard diet ad libitum (17.5% crude protein, 2.3% ether extract, and 16.8% crude fibre, at 2600 Kcal DE/Kg, NANTA S.A., Valencia, Spain) and had free access to water.

### 2.4. Semen Collection, Evaluation, and Preparation of Seminal Doses

Semen from 12 adult males was used. Two ejaculates per male were collected with a minimum of 30 min between ejaculate collections, on a single day, using an artificial vagina. A subjective sperm evaluation was performed to assess the initial seminal quality. Only ejaculates exhibiting a white colour and possessing more than a 70% motility rate, 85% normal intact acrosome, and less than 15% abnormal sperm were pooled and used in this experiment. All other ejaculates were discarded.

Seminal pools were first diluted in a ratio of 1:2 (vol/vol) with the control medium, then split into three equal fractions and diluted in a ratio of 1:5 with one of the three experimental extenders in order to obtain a final GnRH concentration of 8 μg/mL with CS—SD and CS—ALG extenders, and with no GnRH for Control group.

After the insemination procedure, the seminal quality of an aliquot of each experimental extender was evaluated. A 20 µL aliquot was diluted in a ratio of 1:50 with 0.25% glutaraldehyde in a phosphate-buffered saline solution to calculate the concentration in a Thoma chamber and to evaluate the percentages of spermatozoa with a normal apical ridge via phase contrast at a magnification of 400×.

The motility characteristics were assessed as described by Viudes de Castro et al. [6] using a computer-assisted sperm analysis system (ISAS, version 1.0.17, Proiser, Valencia, Spain) operating at 30 video frames per second (30 Hz), with the setting of a particle area from 20 to 80 mm. A sperm with an average path velocity (VAP) of less than 10 mm/sec were considered non-motile, while the sperm that presented a VAP greater than 50 mm and/or a straightness index of >70% was considered progressively motile. To assess motility parameters, the sperm sample concentration was adjusted to 7.5 × 10^6^ sperm/mL with a Tris-citric acid-glucose extender (TCG) supplemented with 2 g/L of bovine serum albumin (BSA). Diluted samples were placed into a Makler counting chamber (Sefi Medical Instruments, Haifa, Israel) and motility characteristics from at least four fields were assessed at 37 °C via negative-phase-contrast optics at a magnification of 100× (NIKON Eclipse 90i microscope, Nikon Corporation Instruments Company; IZASA, Barcelona, Spain) connected to the computer through a monochrome Basler A312f video camera (Basler AG, Ahrensburg, Germany). Individual sperm tracks were visually assessed to eliminate possible debris and misdiagnosed tracks.

The percentage of viable sperm was determined via flow cytometry. All the dilutions were performed at room temperature. Samples were diluted to 30 × 10^6^ sperm/mL with the TCG extender supplemented with 0.2% BSA. The percentage of viable sperm was determined using dual fluorescent staining with SYBR-14/PI [6]. An aliquot of 100 μL of the diluted sample was transferred into a tube containing 0.45 mL of TCG, 2.5 μL of SYBR-14 (10 mM solution in DMSO), and 2.5 μL of PI (1.5 mM solution in purified water). Then, the sample was filtered through a 40 mm nylon mesh to remove large clump of cells and debris and incubated at room temperature for 10 min in the dark. Flow cytometry analyses were performed using a Coulter Epics XL cytometer (Beckman Coulter, IZASA, Barcelona, Spain) equipped with standard optics (a 15-mW488 nm argon ion laser; Cyonics; Coherent, Santa Clara, CA, USA). The green fluorescence of SYBR-14 was detected using a 550 nm long-pass filter combined with a 525 nm (bandwidth 505–545) band-pass filter (FL1). The red fluorescence of PI was detected using a 645 nm long-pass filter combined with a 620 nm (bandwidth 605–635) band-pass filter (FL3). A total of 10,000 gated events (based on the forward and side scatter of the sperm population recorded in the linear mode) were considered per sample. Flow cytometry data were analysed with the software Expo32ADC (Beckman Coulter Inc.). Only the percentages of live sperm were considered in the results (SYBR-14-positive and PI-negative).

### 2.5. Insemination Procedure

All does used in this experiment were non-lactating females with at least three delivered births. In order to achieve the same high receptivity rate, all females received an intramuscular injection of 20 IU of eCG (Cuniser 500, Laboratorios Hipra S.A., Gerona, Spain) two days before insemination. A total of 356 inseminations were performed in seven batches. Females were inseminated with 0.5 mL of diluted semen using standard curved cannulas (24 cm). Each female was randomly assigned to one of the three experimental groups (Table 1):

Fertility rate at birth (number of does giving birth/number of inseminated does) and prolificacy (number of total and number of liveborns kits per litter) were the parameters considered for reproductive performance at birth.

### 2.6. Statistical Analysis

A GLM including the group as a fixed effect was performed with the SPSS 16.0 software package (SPSS Inc., Chicago, IL, USA, 2002). Shapiro–Wilk tests were conducted in the SPSS Explore procedure to assess the normality of the residuals (Gaussian distribution) and the homogeneity of variances was evaluated using the Levene test. For semen quality traits (motility, viability, and acrosome integrity), EE and prolificacy, an ANOVA was used, whereas for the fertility rate at birth, a probit link with binomial error distribution was used. Differences between groups were assessed using Bonferroni’s test. Values were considered statistically different at *p* < 0.05. Data are expressed as the least-squares mean ± standard error of means (LSM ± SEM).

## 3. Results

The extender used had no effect on semen quality parameters. The pools used in the present study presented an average sperm concentration of 354 × 10^6^ sperm/mL. The motility, viability and acrosome integrity were similar among groups (81.2 ± 7.9% total motility; 51.2 ± 9.1% progressive motility; 68.9 ± 4.80% viability; 89.1 ± 2.5% acrosome integrity.

As shown in Table 2, although the initial EE was higher than 90% in both encapsulation systems, the value observed for the CS—DS was significantly higher than that for CS—ALG. Five hours later, after incubation at 5 °C, both systems showed similar EE results to those at initial entrapment. However, after 5 h of incubation at 37 °C, the EE in both systems decreased significantly between 8 and 10% with respect to the initial EE, showing that the CS—DS complex had significantly higher values of EE than CS—ALG did.

Fertility rate at birth and prolificacy values are presented in Table 3. Results of the in vivo study showed that females inseminated with both GnRH-analogue protection systems (CS—DS and CS—ALG groups) achieved a reproductive performance similar to that of the control group. No significant differences in fertility rate at birth and prolificacy were observed between intravaginal and intramuscular administrations of the GnRH analogue (Table 3).

## 4. Discussion

In rabbit AI, one welfare-oriented method with which to induce ovulation is the use of extenders supplemented with GnRH analogues. In this study, the efficacy of two CS-based encapsulation systems at entrapping the GnRH analogue used, CS—DS and CS—ALG complexes, and their effect on reproductive performance were investigated. Intravaginal administration of GnRH analogues is a less stressful procedure with which to induce ovulation than an intramuscular injection is and represents a refinement in the zootechnical practice of AI in this species. Unfortunately, degradation of a GnRH analogue when exposed to enzymes present in seminal plasma and its poor permeation through vaginal mucosa limits its bioavailability and, consequently, it is necessary to increase the GnRH analogue concentration. Peptide instability is the main reason why GnRH analogues are traditionally administered through injection rather than intravaginally. There is a great need to enhance the absorption of GnRH analogues in order to decrease the concentration of the hormone used in rabbit AI extenders. Nanocarriers could protect GnRH analogues from proteolytic activity, improving their bioavailability. Consequently, this would allow a reduction in the required doses of analogues in insemination extenders to effectively induce ovulation. It has been shown that mixing oppositely charged polyelectrolytes in solution will result in their self-assembly due to the formation of electrostatic links. These interactions between the polymeric chains lead to drug encapsulation avoiding the use of covalent cross-linkers. CS is one of the most popular biopolymers for drug nanoencapsulation development, and CS-based complexes with natural polymers have received much attention in recent years, as they are ideal candidates for the transport and release of bioactive molecules in pharmaceutical applications, especially for sensitive biological molecules, preserving their integrity and properties [27]. ALG is one of the most commonly studied anionic polyelectrolytes in complexation with CS for drug encapsulation [20]. In previous studies, we have shown that the presence of aminopeptidases inhibitors bestatin and EDTA in semen extenders supplemented with buserelin acetate did not affect the reproductive performance of does [15]. Recently, we proved that it was possible to reduce the analogue concentration used in the extender by 20% when CS—DS nanoparticles were used as a carrier for this GnRH analogue [24]. In the present study, the encapsulation efficiency for the CS—DS complex was higher than that previously observed. It has been proposed that stirring speed and time affect entrapment efficiency [28]. Therefore, it seems that modifications carried out in the preparation of nanoparticles in the present work increased the entrapment efficiency. Both nanoparticle systems used in the present work would protect almost 90% of the buserelin acetate. This encapsulation efficiency is comparable to that observed by other authors [29,30] working with proteins encapsulated in CS—DS nanoparticles, and with a GnRH analogue encapsulated into CS–sodium tripolyphosphate (TPP) [25]. Although CS—SD nanoparticles showed greater entrapment efficiency than CS—ALG nanoparticles did, the use of both resulted in similar reproductive performances, and both groups reached the fertility rate observed in females treated intramuscularly with buserelin acetate. However, these results differ from the findings of Hassanein et al. [25], who showed that the timing of the preovulatory LH surge in does treated intravaginally with 4 μg of buserelin acetate loaded in CS—TPP nanoparticles was earlier than that observed in the control group (does treated intramuscularly), and that treatment with 2 μg of buserelin acetate loaded in CS—TPP nanoparticles failed to induce the female LH surge. They observed lower reproductive performance in rabbit does induced to ovulate via the vaginal route with 4 μg of buserelin acetate loaded in CS—TPP nanoparticles compared to that of intramuscularly treated females. This discrepancy must be due in part to the drug-loading system used during the preparation of the nanoparticles. In the present work, the GnRH analogue was incorporated into the CS complex and, after 5 h of incubation at 37 °C, the EE in both systems was between 8 and 10% with respect to the initial EE, while Hassanein et al. [25] loaded the GnRH analogue onto the surface of the nanoparticles, and after 5 h of incubation at 37 °C, the release of the GnRH analogue reached approximately 30%. Therefore, it is possible that the hormone encapsulation system with CS—DS and CS—ALG protects the GnRH analogue more efficiently than does hormonal adsorption on the surface of CS—TPP nanoparticles. As previously mentioned, another way to increase the bioavailability of GnRH is the use of absorption enhancers. Therefore, it is likely that an epithelial permeation-enhancing action was present in the diluents used in the present work due to the presence of EDTA. As a chelating-type penetration enhancer, the interaction between the EDTA and the Ca^2+^ ions in the epithelial surface can cause a paracellular and transcellular increase in transport [19], improving the absorption of the GnRH analogue and enhancing its efficacy.

The present findings suggest that the use of the CS—DS and CS—ALG complexes as GnRH carriers allow a reduction in hormone concentration in the seminal dose, without affecting the fertility or prolificacy of females. Hence, each nanoencapsulation system presented here is an efficient method of intravaginal ovulation induction, allowing a reduction in the concentration of the GnRH analogue in seminal doses. Therefore, this system provides greater animal welfare, improves biological safety in the use of endocrine products and reduces costs. Furthermore, seminal doses in this species are prepared for use between 2 and 36 h after their dilution. Therefore, more studies on nanoparticle-entrapped GnRH analogues should be performed to determine whether or not it is possible to further reduce the concentration of the GnRH analogue used in extenders and to determine their efficiency during long storage periods.

## 5. Conclusions

Chitosan-based nanoparticles as carriers of buserelin acetate in rabbit insemination extenders can enhance the bioavailability of the analogue used, allowing a reduction in the hormonal concentration added to semen extender without affecting the reproductive performance of does.

## Figures and Tables

**Table 1:** Experimental groups description.

Group	Description
Control	Does inseminated with 0.5 mL of diluted semen in control extender. At the time of insemination, females were treated intramuscularly with 1 µg of buserelin acetate to induce ovulation.
CS—DS	Does inseminated with 0.5 mL of diluted semen in extender with GnRH loaded in CS/DS nanoparticles (4 μg buserelin acetate/doe).
CS—ALG	Does inseminated with 0.5 mL of diluted semen in extender with GnRH loaded in CS/ALG nanoparticles (4 μg buserelin acetate/doe).

**Table 2:** Entrapment efficiency of busereline acetate (EE%. Data are expressed as least-squares means ± standard error).

Carrier System	0 h	After 5 h at 5 °C	After 5 h at 37 °C
CS—DS	96.0 ± 0.13 ^a^	96.5 ± 0.68 ^a^	90.9 ± 0.40 ^a^
CS—ALG	90.4 ± 0.21 ^b^	89.6 ± 0.01 ^b^	82.9 ± 0.03 ^b^

CS—DS: chitosan–dextran sulphate; CS—ALG: chitosan–alginate. Values within a column with different superscripts differ at *p* < 0.05.

**Table 3:** Reproductive performance of female rabbits according to treatment.

Group	N	Fertility at Birth	TB	BA
CS—DS	142	0.68 ± 0.040	9.4 ± 0.35	8.0 ± 0.39
CS—ALG	144	0.69 ± 0.042	9.7 ± 0.37	8.9 ± 0.41
Control	70	0.73 ± 0.081	10.6 ± 0.75	9.2 ± 0.84

TB: Total number of kits born per litter; BA: number of liveborn kits per litter. CS—DS: chitosan–dextran sulphate; CS—ALG: chitosan–alginate.

## Data Availability

The original contributions presented in the study are included in the article, and further inquiries can be directed to the corresponding authors.
